# Radiation dose enhancement using gold nanoparticles with a diamond linear accelerator target: a multiple cell type analysis

**DOI:** 10.1038/s41598-022-05339-z

**Published:** 2022-01-28

**Authors:** Olivia Piccolo, John D. Lincoln, Nicole Melong, Benno C. Orr, Nicholas R. Fernandez, Jennifer Borsavage, Jason N. Berman, James Robar, Michael N. Ha

**Affiliations:** 1 Department of Biology, Dalhousie University, Halifax, NS Canada; 2 Department of Pediatrics, IWK Health Centre/Dalhousie University, Halifax, NS Canada; 3 Department of Physics and Atmospheric Science, Dalhousie University, Halifax, NS Canada; 4 Children’s Hospital of Eastern Ontario Research Institute/Department of Pediatrics, University of Ottawa, Ottawa, ON Canada; 5 Department of Radiation Oncology, Dalhousie University, Halifax, NS Canada

**Keywords:** Cancer therapy, Radiotherapy, Nanotechnology in cancer, Applied physics

## Abstract

Radiotherapy (RT) is an effective cancer treatment modality, but standard RT often causes collateral damage to nearby healthy tissues. To increase therapeutic ratio, radiosensitization via gold nanoparticles (GNPs) has been shown to be effective. One challenge is that megavoltage beams generated by clinical linear accelerators are poor initiators of the photoelectric effect. Previous computer models predicted that a diamond target beam (DTB) will yield 400% more low-energy photons, increasing the probability of interacting with GNPs to enhance the radiation dose by 7.7-fold in the GNP vicinity. After testing DTB radiation coupled with GNPs in multiple cell types, we demonstrate decreased head-and-neck cancer (HNC) cell viability in vitro and enhanced cell-killing in zebrafish xenografts compared to standard RT. HNC cell lines also displayed increased double-stranded DNA breaks with DTB irradiation in the presence of GNPs. This study presents preclinical responses to GNP-enhanced radiotherapy with the novel DTB, providing the first functional data to support the theoretical evidence for radiosensitization via GNPs in this context, and highlighting the potential of this approach to optimize the efficacy of RT in anatomically difficult-to-treat tumors.

## Introduction

Radiotherapy (RT) is used for approximately 50% of cancer patients^1,2^. Despite being an effective treatment modality, patients who receive RT often experience damage to healthy tissue due to limitations on confining a sufficient radiation dose to the tumor^2,3^. The potential accumulation of toxicities in proximal tissues restricts the optimal therapeutic dose of radiation that can be safely delivered^3^. Improving the therapeutic window would enhance targeted delivery of RT.

High atomic number (Z) nanoparticles, like gold (GNPs) (Z = 79), have the capacity to enhance targeted radiation damage in vitro and in vivo^4–8^. One proposed mechanism by which this occurs is the photoelectric effect, which causes the emissions of short-range Auger electrons and characteristic X-rays^5,7,9^. The probability of photoelectric interactions increases for lower energy photons and materials with higher atomic numbers^5,10–12^. However, a barrier to GNP-enhanced RT is the small proportion of low energy photons in clinical photon beams produced by standard linear accelerators (LINACs). To overcome this challenge, we have developed a novel beam line with a low Z target (Z = 6)^6^. This diamond target beam (DTB) quadruples the proportion of low energy photons at 10 cm depth (Supplemental Fig. S1), increasing the GNPs’ release of short-ranged Auger electrons and characteristic X-rays to magnify the dose^6,13^. Increasing the probability for X-ray interactions is essential to the efficacy of the DTB to induce damage in tumors, and was recently illustrated in a gadolinium nanoparticle-based study using a filter-free LINAC beam^6,10^. Emerging research on NPs illustrates surface functionalization of GNPs to have advantages for biomedical applications such as nuclear imaging, radiometabolic therapy^14–16^ and targeted delivery of compounds like chemotherapeutic drugs to further radiosensitize cancer cells^7^.

The zebrafish (*Danio rerio)* is a well-established animal model to study cancer due to its small size, lack of early adaptive immune system, and high degree of genetic conservation with humans^17–21^. The larval zebrafish xenotransplantation (XT) platform has been effectively employed to study cancer cell proliferation, migration, and therapy response^20,22–25^. This model provides simple experimental conditions to study in vivo responses to the DTB.

Although the physical mechanism behind the radiosensitization reaction between GNPs and the DTB has been theorized^6^, demonstration in biological systems is comparatively sparse. Utilizing a prototype LINAC to generate the DTB, we characterized the biological effects of RT and responses to human cancer cells exposed to GNPs. We demonstrate that GNP-facilitated DTB radiotherapy (DTBR) effectively increases cancer cell killing in vitro, and in xenografted head-and-neck cancer cell (HNCC) lines in zebrafish larvae.

## Results

### GNP uptake and localization in lysosomes is cancer cell type specific

**Figure 1:**
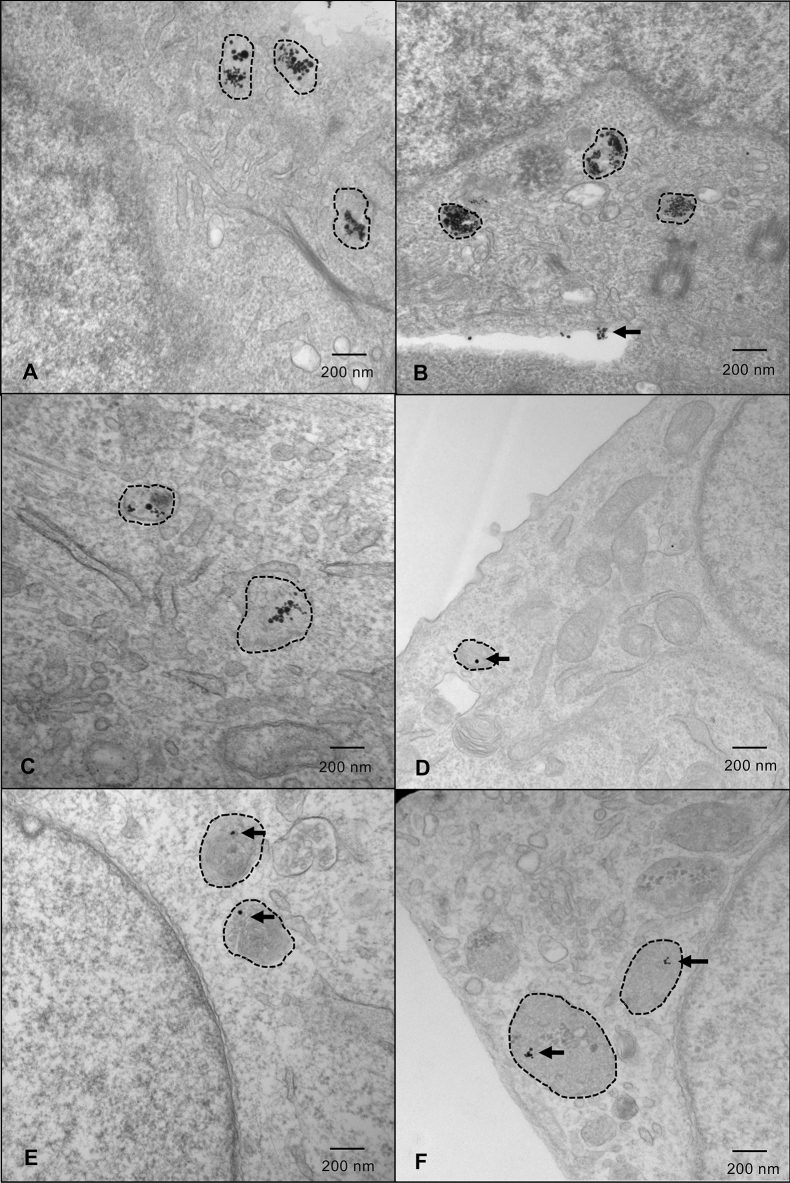
HNC cells demonstrate the greatest uptake of GNPs in lysosomes. Representative images of cells labelled with 25 µM GNPs. Lysosomes are indicated with hashed outlines. (**a**) FaDu; (**b**) HSC-3; (**c**) Detroit-562; (**d**) SK-N-AS; (**e**) Panc1; and (**f**) A673 cells. Cells were fixed approximately 36 h after GNP-labelling (approximate radiation timepoint) and images were captured by a transmission electron microscope. Imaging was done in triplicate—5 images were captured for each sample, with 3 samples/replicate, and 3 replicates in total. *HNC* head and neck cancer, *GNPs* gold nanoparticles.

Diffusion of GNPs into cell lysosomes was verified with Transmission Electron Microscopy (TEM). GNP uptake was highest in FaDu, HSC-3 and Detroit-562 HNCC lines, in a concentration-dependent manner (Fig. 1, Supplemental Figs. S2–S4). In contrast, SK-N-AS neuroblastoma, Panc1 pancreatic adenocarcinoma, and A673 Ewing sarcoma cell lines exhibited minimal uptake of GNPs at 25 µM (Fig. 1). In vitro cell toxicity curves and TEM studies determined 25 µM as the optimal concentration of GNPs (Supplemental Figs. S2, S3). GNPs were observed using TEM in cells as early as 24 h post-labeling and remained in cells for ~ 48 h after labeling (Supplemental Fig. S5).

### GNP-mediated DTB radiotherapy reduced in vitro cell viability and tumorigenic potential in head and neck cancer cells

**Figure 2:**
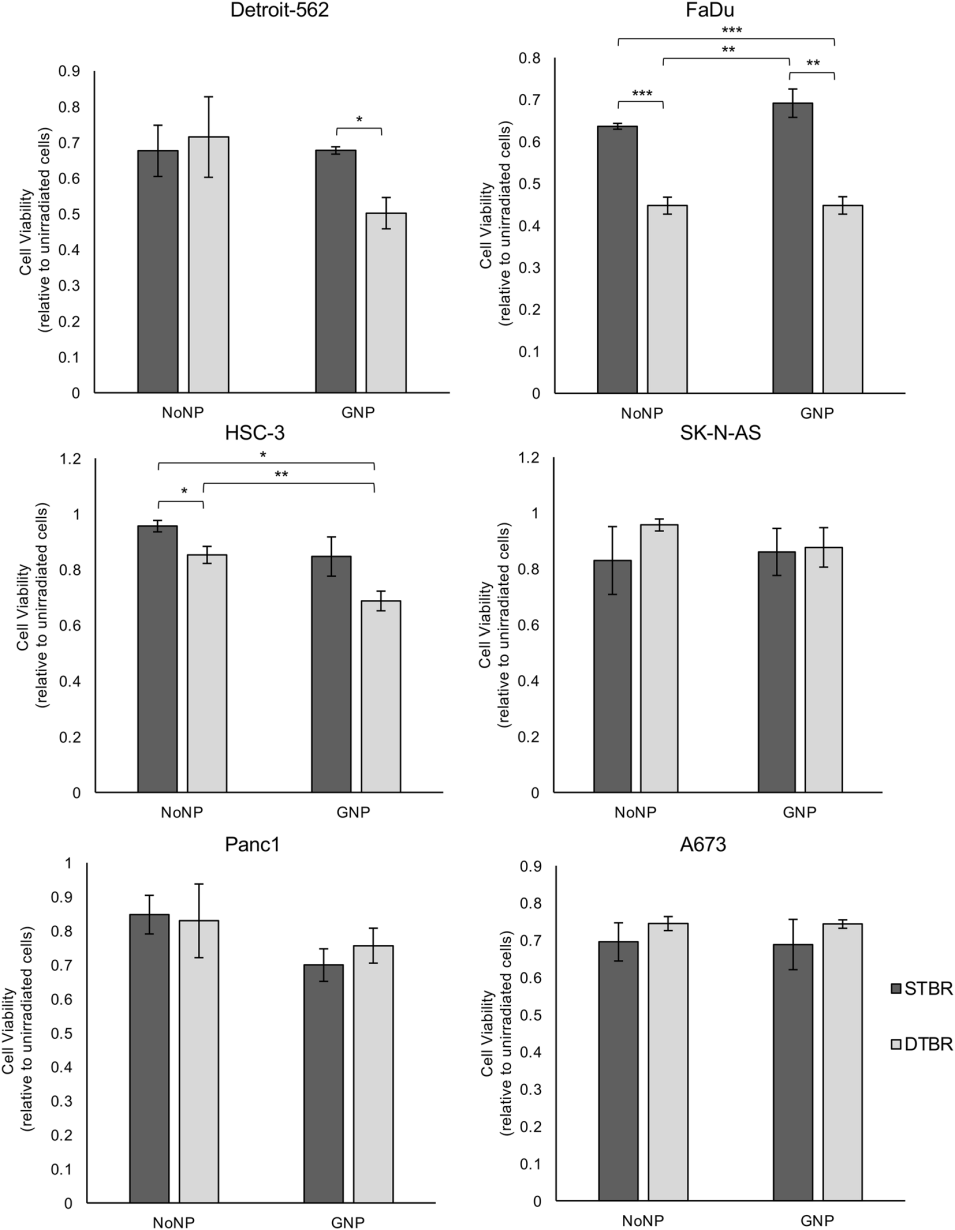
GNP-labeled HNCCs irradiated with the DTB show reduced viability 3 days post-irradiation. The alamarBlue viability assay was used to determine the levels of oxidative phosphorylation in cells irradiated with the STB or DTB and labelled with GNPs or NoNPs, 3 days after radiation. Detroit-562, FaDu, and HSC-3 HNCC lines demonstrated a significant reduction in cell viability following 8 Gy irradiation with the DTB in cells labelled with GNP compared to NoNP-labelled cells with STBR (p = 0.018, p = 0.004, p = 0.003, respectively). NoNP FaDu cells irradiated with the DTB demonstrated decreased viability compared to NoNP and GNP cells with STBR (p = 0.001 and p = 0.003, respectively). Similarly, NoNP HSC-3 cells irradiated with the DTB demonstrated a reduction in viability compared to NoNP cells with STBR (p = 0.049). With DTBR, GNP-labelled HSC-3 cells decreased compared to NoNP cells (p = 0.024). SK-N-AS cells, Panc1 cells, and A673 cells demonstrated no significant differences in viability. Cell viability values are made relative to control cells that were not irradiated and are presented as the fold change means ± standard error of the mean, p* < 0.05, p** < 0.01, p*** < 0.001 for significant decrease in cell viability. Significance between groups was tested using one-way analysis of variance (ANOVA) with a Tukey multiple comparisons test (n = 3; 10 wells per group/replicate). *GNP* gold nanoparticles, *NoNP* no nanoparticles, *HNCC* head and neck cancer cells, *STB(R)* standard target beam (radiation), *DTB(R)* diamond target beam (radiation).

alamarBlue assays were used to assess in vitro cell viability 3 days following treatment with the DTBR. Colony formation assays (CFA) were used to measure tumorigenicity by measuring the surviving fraction of cells 10 days after radiation. Panc1, SK-N-AS, A673 cells, and the three HNCC lines (FaDu, HSC-3, Detroit-562) were examined for in vitro responses to GNP-mediated DTBR with the alamarBlue assay. Detroit-562, FaDu, and HSC-3 cell viability was reduced by 17.4%, 18.9%, and 26.8%, respectively, with GNP-DTBR compared to No nanoparticle (NP) cells irradiated with the standard target beam (STB) (p = 0.018, p = 0.004, and p = 0.003, respectively, Fig. 2). SK-N-AS, Panc1, and A673 cell survival did not differ significantly in response to the presence of GNP or to the type of beam target (Fig. 2).

**Figure 3:**
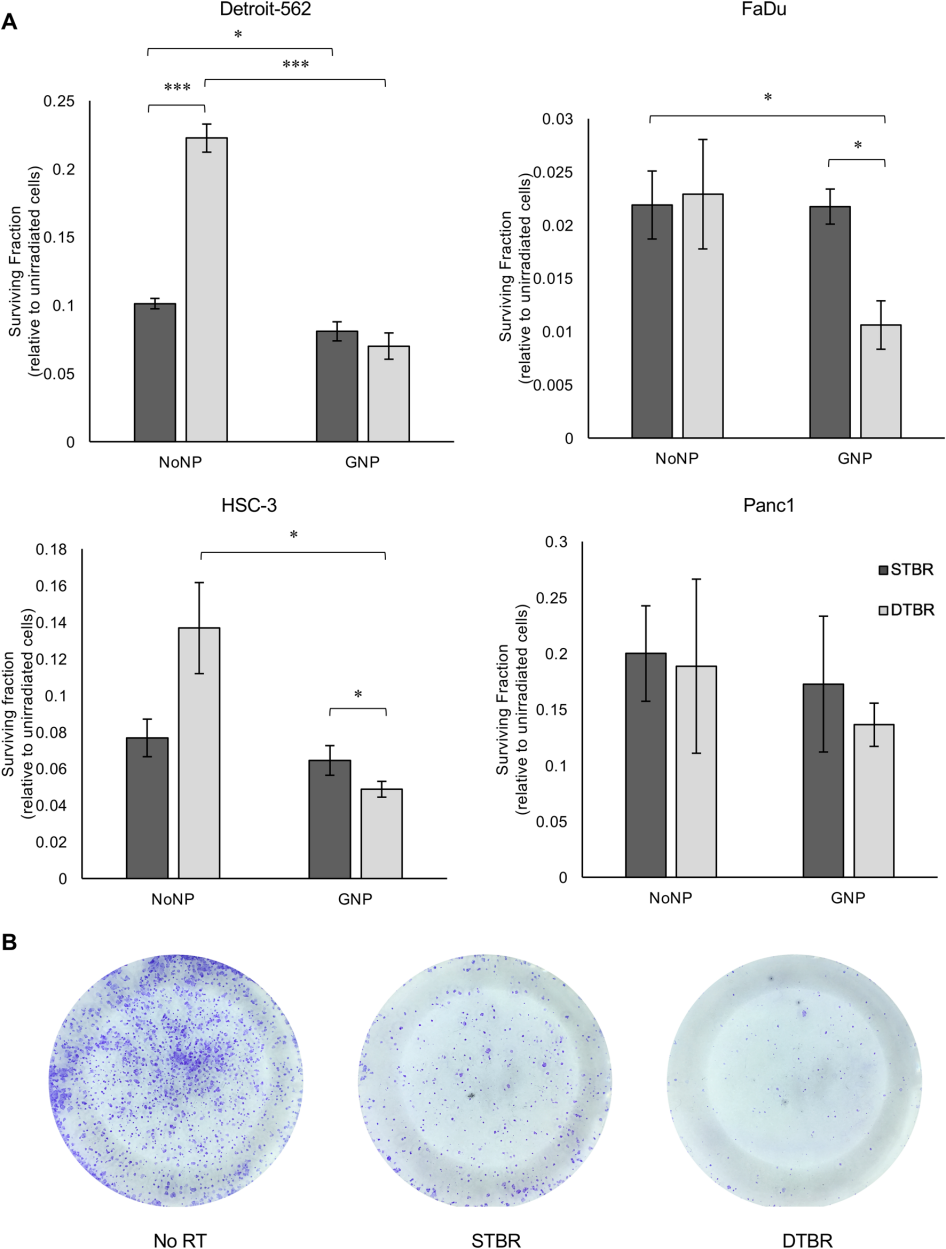
HNCCs labelled with GNPs show decreased in vitro colony forming potential 10 days post radiation. A colony formation assay (CFA) was used to determine the potential of GNP-labelled or unlabelled (NoNP) cancer cells to form colonies 10 days after radiation with the STB or DTB. (**a**) GNP-labeled Detroit-562 and FaDu cells irradiated with the DTB exhibit decreased colony formation potential compared to NoNP-labeled cells with STBR (both p = 0.045). With DTBR, GNP-labeled Detroit-562 cells formed fewer colonies than NoNP-labeled cells (p = 0.0001). GNP-labeled FaDu cells also demonstrated decreased colony forming potential with DTBR compared to STBR (p = 0.017). GNP-labelled HSC-3 cells formed fewer colonies with both DTB and STB radiation compared to unlabeled (NoNP) cells treated with DTBR (p = 0.025 and p = 0.045, respectively). Lastly, with NoNPs, STBR treated Detroit-562 cells formed fewer colonies than cells with DTBR (p = 0.0001). Panc1 cells exhibited a similar trend in the reduction of colonies in GNP-labeled groups with DTBR compared to other treatment groups, but the results were not significant. (**b**) Representative bright-field images of GNP-labeled HSC-3 cells fixed and stained with Crystal Violet in Petri plates receiving no radiation (No RT), STBR or DTBR. Results are presented as the fold change of surviving fraction (SF) means ± standard error of the mean, SF = (# of colonies/(plating efficiency (PE) × # of colonies seeded)) × 100. Fold changes were made relative to unirradiated control cells, p* < 0.05, p** < 0.01, p*** < 0.001 for significant decreases in colony forming potential. Significance between groups was tested using one-way analysis of variance (ANOVA) with a Tukey multiple comparisons test (n = 3). *GNP* gold nanoparticles, *NoNP* no nanoparticles, *HNCC* head and neck cancer cells, *STB(R)* standard target beam (radiation), *DTB(R)* diamond target beam (radiation).

To validate these in vitro responses and assess more long-term effects, a CFA was used to assess cell tumorigenic potential 10 days after 8 Gy radiation. In CFA experiments, only HNCC lines were investigated due to their significant uptake of GNPs and response to GNP-mediated DTBR. Panc1 cells were included as a control after establishing limited uptake of GNPs and observing no significant reduction in the surviving fraction of cells. In FaDu and HSC-3 cells, DTB selectively enhanced the GNP-mediated decrease in tumorigenic potential compared to cells with No NPs irradiated with the DTB (p = 0.017 and 0.05, respectively). In Detroit-562 and FaDu cells, the combination of GNPs and DTBR decreased colony formation compared to No NP irradiated with the STB (p = 0.0001 and 0.045, respectively, Fig. 3).

### GNP-DTB radiotherapy enhanced HNCC killing in vivo

**Figure 4:**
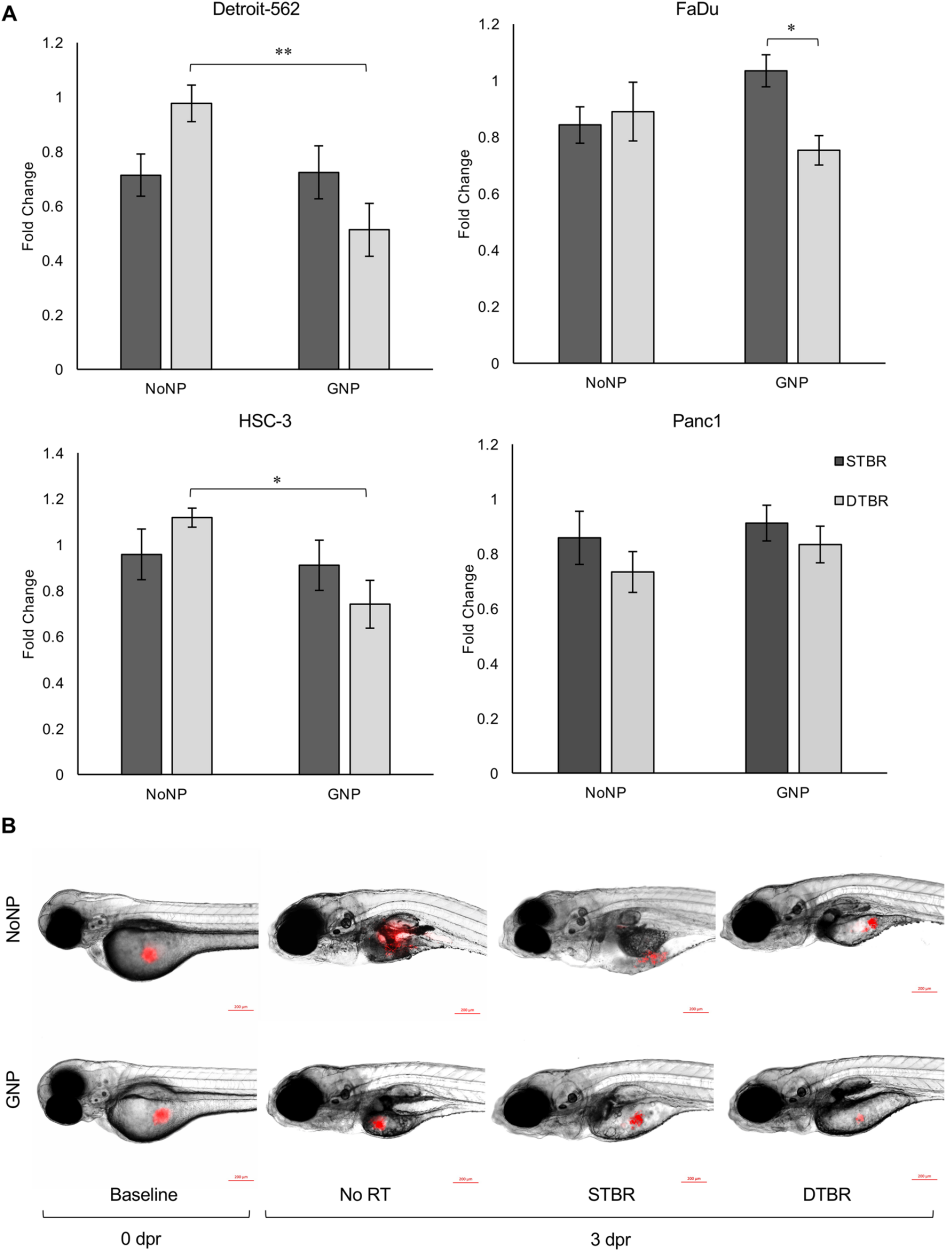
GNP-mediated DTBR significantly decreased HNCC proliferation in vivo. Groups of 15–20 zebrafish larvae were used for each time point and treatment group, 50–100 cells were injected into the yolk sac of each fish, and the number of fluorescent cells was quantified ex vivo. Xenografted cells were quantified at baseline (0 days post radiation (dpr)) and 3 dpr after treatment with 8 Gy from the STB or DTB. Results are represented as fold change of the unirradiated control cells (No RT). (**a**) With GNP-DTBR, Detroit-562 and HSC-3 xenografted cells demonstrate increased cell death compared to NoNP-DTBR (p = 0.005 and p = 0.015, respectively), and FaDu cells exhibit increased cell death compared to GNP-STBR (p = 0.022). There were no significant differences in Panc1 cancer cell proliferation between treatment groups in vivo. (**b**) Representative images of fluorescently labelled HSC-3 HNCCs injected into the yolk sac of *casper* larvae, scale bars are 200 µM. Results are presented as fold change means ± standard error of the mean, p* < 0.05, p** < 0.01, p*** < 0.001 for significant decreases in in vivo cell proliferation. Significance between groups was tested using one-way analysis of variance (ANOVA) with a Tukey multiple comparisons test (n = 3; 20 larvae per group/replicate). *GNP* gold nanoparticles, *NoNP* no nanoparticles, *HNCC* head and neck cancer cells, *STB(R)* standard target beam (radiation), *DTB(R)* diamond target beam (radiation).

To further validate the observed in vitro responses, we employed an in vivo xenotransplantation model. Detroit-562 and HSC-3 cells engrafted and proliferated in zebrafish larvae in unirradiated control groups, but FaDu and Panc1 cells did not. Despite these cells not proliferating, we still undertook a comparative analysis on the cell killing potential of the novel DTB to that of the STB. With DTBR, GNPs significantly increased cell death in Detroit-562 and HSC-3 cells compared to cells with no NPs (p = 0.005 and p = 0.015, respectively, Fig. 4).

### DTB radiation increased levels of reactive oxygen species and double strand breaks in GNP-labeled HNCCs

RT directly causes cell death by DNA double strand breaks (DSBs) and indirectly by the production of reactive oxygen species (ROS)^26^. To determine whether GNP-facilitated DTBR directly disturbs DNA integrity, γ-H2AX monoclonal antibody and DAPI nuclear stain were used. H2AX is a histone, phosphorylated on Ser-139 to form γ-H2AX foci in response to DNA DSBs^27,28^.

**Figure 5:**
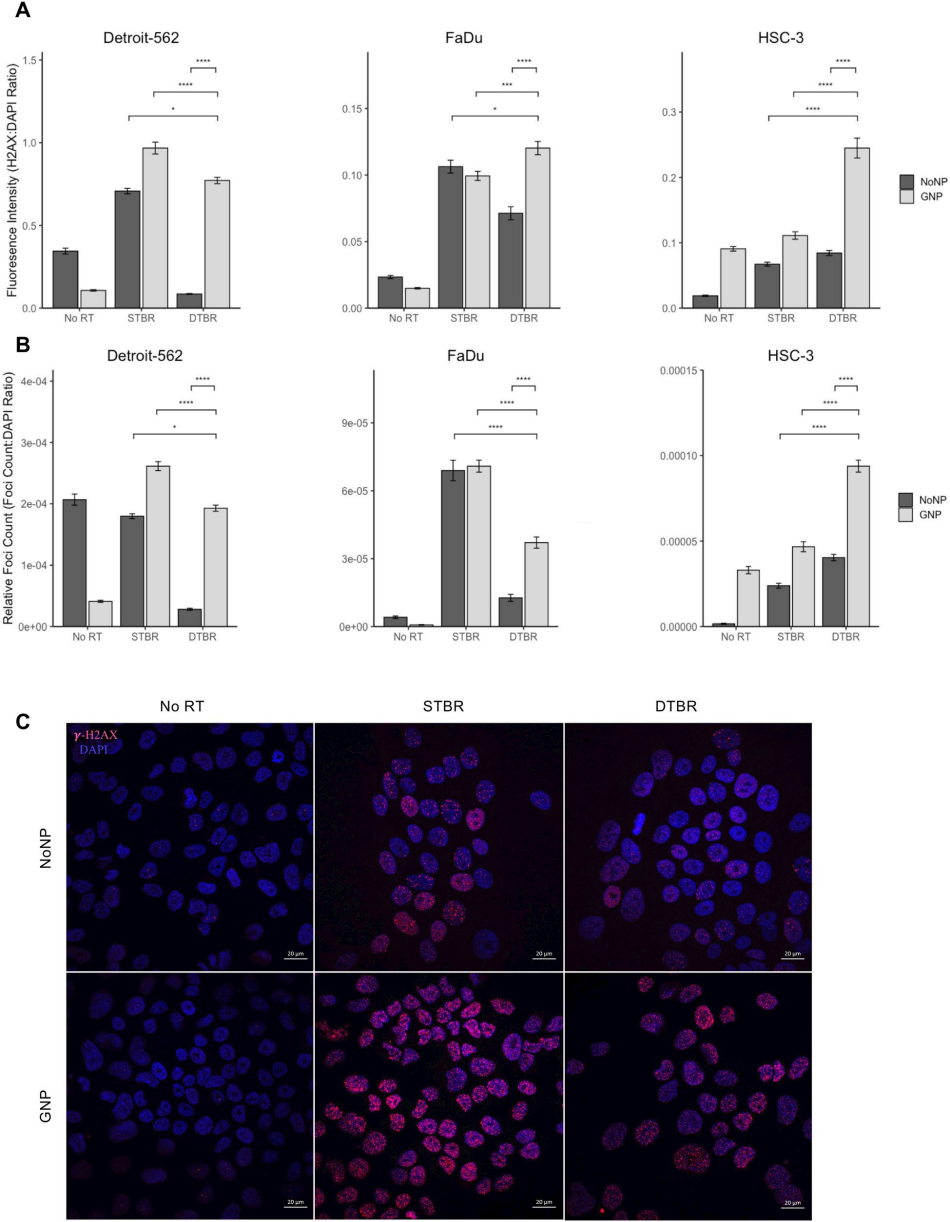
GNP labelled Detroit-562 and HSC-3 HNCCs demonstrate increased fluorescence intensity (FI) of double stranded breaks (DSBs) with DTBR compared to NoNP cells with STBR. Cells were cultured in 6-well plates, irradiated with 8 Gy from the STB or DTB, then fixed 30 min after radiation. Cells were processed for immunohistochemistry (IHC) with γ-H2AX to assess DNA double strand breaks and DAPI nuclear stain. (**a**) The FI of γ-H2AX foci was determined and measured relative to the FI of DAPI nuclear stain within each nucleus. GNP-labeled Detroit-562 and HSC-3 cells that received DTBR demonstrated increased FI of γ-H2AX foci than No NP cells with STBR (p = 0.049 and p < 0.0001, respectively). With DTBR, Detroit-562, FaDu, and HSC-3 GNP-labeled cells demonstrated greater FI of γ-H2AX foci compared to No NPs (p < 0.0001 for all). GNP-labeled FaDu and HSC-3 cells exhibited increased FI with DTBR compared to STBR (p = 0.001 and p < 0.0001, respectively), but GNP-labeled Detroit-562 cells displayed decreased FI with DTBR compared to STBR (p < 0.0001). (**b**) The number of γ-H2AX foci was analysed and made relative to the nuclear area. With DTBR, GNP-labeled Detroit-562, FaDu, and HSC-3 cells exhibited significantly greater foci/nucleus than No NP cells (p < 0.0001, p < 0.0001, and p < 0.001, respectively). GNP-labeled Detroit-562 and HSC-3 cells that received DTBR display greater foci/nucleus than No NP cells with STBR (p = 0.0397 and p < 0.0001, respectively), but GNP-labeled FaDu cells with DTBR demonstrated a decrease in foci/nucleus compared to No NP cells with STBR (p < 0.0001). Lastly, GNP-labeled Detroit-562 and FaDu cells with DTBR displayed significantly less foci/nucleus than with STBR (p < 0.0001 for both), but HSC-3 cells exhibited more (p < 0.0001). (**c**) Representative confocal images of Detroit-562 cells post RT (Zeiss LSM 710 Laser Scanning Confocal microscope at 40 ×). Scale bars = 20 μm. Results are presented as means of [a] Relative fluorescence intensity and [b] Relative Foci count ± standard error of the mean, p* < 0.05, p** < 0.01, p*** < 0.001, p**** < 0.0001. Significance between groups was tested using a standard t-test with Tukey multiple comparison test (n = 3; 4 images/sample). *GNP* gold nanoparticles, *NoNP* no nanoparticles, *HNCC* head and neck cancer cells, *STB(R)* standard target beam (radiation), *DTB(R)* diamond target beam (radiation).

**Figure 6:**
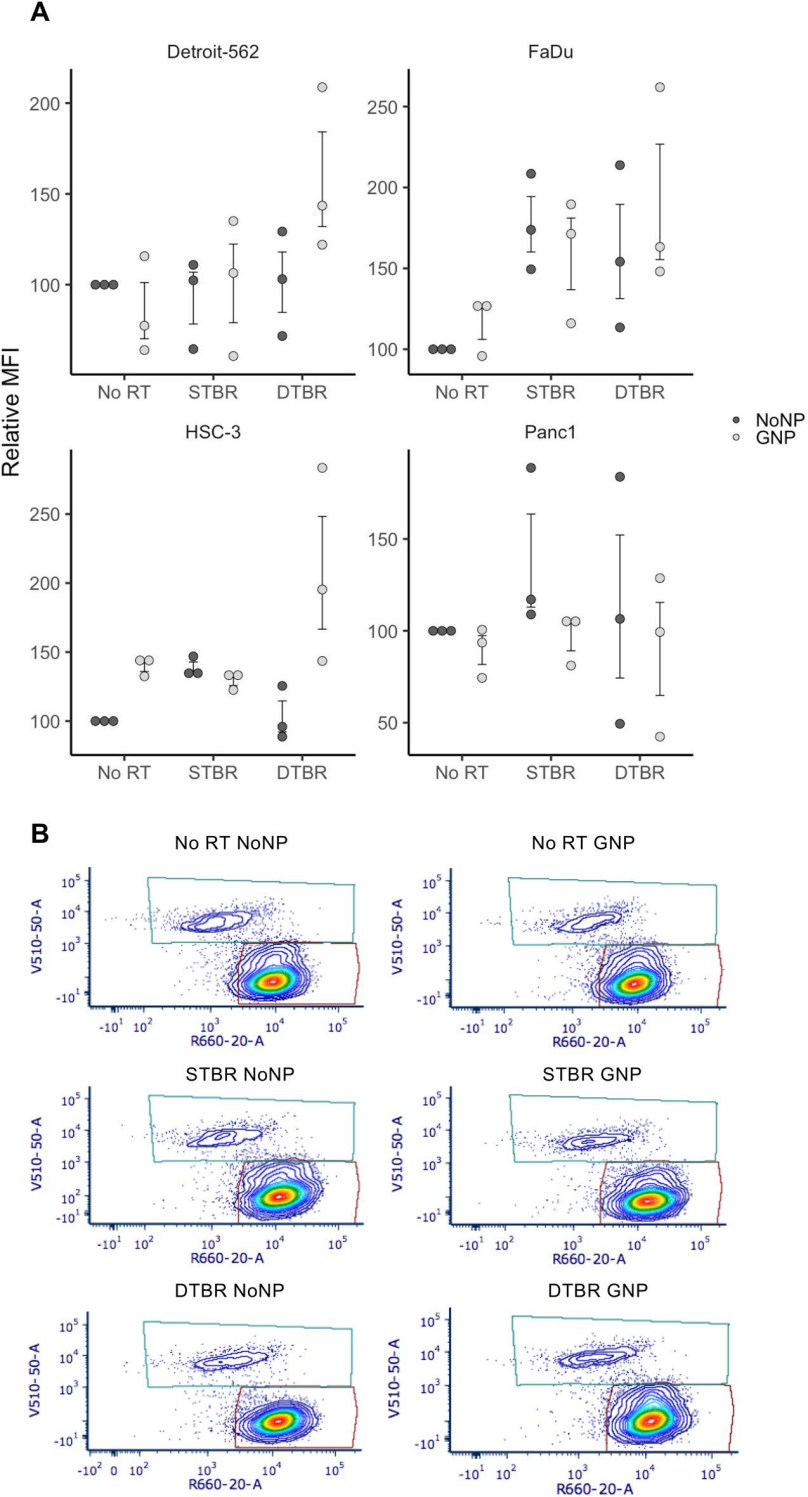
Reactive oxygen species (ROS) were elevated in HNCCs with GNP-facilitated DTBR. Cells were cultured in 6-well plates, irradiated with 8 Gy from the STB or DTB, then processed for flow cytometry 12 h post radiation. (**a**) Detroit-562, FaDu, and HSC-3 HNCC lines showed a trend towards higher levels of ROS in GNP-labeled cells irradiated with the DTB (these increases were not significant). GNP Panc1 cells demonstrated a reduced level of ROS after DTBR. (**b**) Representative flow plots of Detroit-562 cells labelled with CellROX Deep Red Reagent (Ex/Em: 644/665) and SYTOX Blue dead cell stain (Ex/Em: 444/480). Results are presented as means of Relative Median fluorescence intensity (MFI) (where relative MFI = net MFI of treated sample/net MFI of untreated sample × 100) ± standard error of the mean, p* < 0.05, p** < 0.01, p*** < 0.001. Significance between groups was tested using a Mann Whitney test (n = 3; ~ 1 × 10^6^ cells per replicate). *GNP* gold nanoparticles, *NoNP* no nanoparticles, *HNCC* head and neck cancer cells, *STB(R)* standard target beam (radiation), *DTB(R)* diamond target beam (radiation).

The number of γ-H2AX foci, and the average fluorescence intensity (FI) of those foci was measured to assess the quantity and intensity of DSBs. The average FI of γ-H2AX foci in GNP-labelled FaDu and HSC-3 cells irradiated with DTBR was higher than the FI in cells irradiated with STBR (p = 0.014 and p < 0.0001, respectively). In Detroit-562 and HSC-3 cells, there was a significant increase in the FI of GNP labeled, DTB irradiated samples compared to No NP irradiated with STB (p = 0.0049 and p < 0.0001, respectively, Fig. 5a). In all cases, the presence of GNPs increased FI in DTB irradiated cells (p < 0.0001, Fig. 5a). Also, Detroit-562 and HSC-3 cells labeled with GNPs and irradiated with DTB had significantly more DSBs than No NP cells that received radiation with the STB (p = 0.0397 and p < 0.0001, respectively, Fig. 5b). GNP-labeled Detroit-562 and FaDu cells irradiated with the DTB had significantly fewer DSBs than cells irradiated with STB (p < 0.0001 for both, Fig. 5b); while GNP-labeled HSC-3 cells had significantly more γ- H2AX foci/nucleus (DSBs/nucleus) (p < 0.0001, Fig. 5b). Reactive oxygen species (ROS) are produced from photon interaction with water molecules and are responsible for approximately two thirds of cell radiation damage^26^. At 12 h post radiation (hpr), HNCCs labeled with GNPs and treated with DTBR demonstrated a trend of increased ROS production, in the form of superoxide anions, hydroxyl radicals, and hydrogen peroxides, indicated by higher relative median fluorescence intensity (rMFI) compared with other groups (Fig. 6).

## Discussion

Head and neck squamous cell carcinoma (HNC) is the sixth most common cancer worldwide^12,29^. RT is a component in aggressive multimodal treatment regimens for HNC, and is offered to approximately 50% of all patients^2^. Although generally efficacious, patients undergoing RT for HNC often experience various side effects (e.g. xerostomia, mucositis)^2,12,29^. The proximity of tumors to critical organs, and large radiation fields necessary to enhance local tumor control in HNC patients, is a significant hurdle to delivering effective treatment. The significant side effects experienced by some patients receiving RT for HNC indicates substantial room for improvement in modern radiation therapies^2^ to achieve a more favorable therapeutic ratio. Other anatomically hard-to-access tumors, like those seen in pancreatic cancer and sarcomas, often present similar challenges when treated with radiation^30,31^.

The DTB is a novel modification of a conventional LINAC producing a significantly modified X-ray energy spectrum compared to standard therapeutic beams^6,32,33^. This report represents the initial functional characterization of biological effects triggered by DTBR. We have demonstrated the utility and potential of GNP-facilitated DTBR to decrease cell viability and tumorigenic potential of HNCC in vitro and in vivo. Conversely, cell lines derived from pancreatic adenocarcinoma, Ewing sarcoma, and neuroblastoma did not respond to GNP-mediated DTBR. NP-diffusion is dependent on cell-specific structural proteins and adhesion properties^34^, and the positive outcomes observed in HNCC are likely attributable to their successful uptake of GNPs as was observed using TEM (Fig. 1, Supplemental Figs. S3, S5). This finding predicts sparing of cells that do not uptake NPs and endows specificity of the DTBR. Since inadequate GNP uptake could be a barrier to effective DTBR, functionalized molecular targeting of NPs to increase distribution and duration in cells is predicted to further enhance and expand the kill capability of DTBR^12,34^. Notably, GNP-mediated DTBR maintained efficacy in the FaDu cell line, a well-characterized radioresistant HNCC^35^. Furthermore, future research aims to explore the efficacy of the novel DTB radiotherapy in other cell types that have been deemed resistant to standard 6 MV radiation. Although we have demonstrated this therapy to be most effective in HNCCs, DTBR may also be successful in other types of cancer where the intake and localization of NPs is successful.

We have demonstrated that DTBR is effective when used with 15 nm GNPs, which were selected to enhance the dose around the GNP without compromising size-dependent uptake. NP uptake, concentration, and radio-sensitization result from the competition between thermodynamic driving forces and diffusion kinetics, which all depend greatly on NP size^5,34^. In vitro studies exploring various sizes of NPs have observed that GNPs of ~ 50 nm had the greatest uptake^36^. Despite this, our results show efficacy of 15 nm GNP labeling and radiosensitization in an in vivo model. This discrepancy may be due to the overlapping cellular structure of the labeled cell bolus, which allows a volumetric enhancement of deposited dose due to the cells lying on top of each other compared to the relatively orderly structure of a cell monolayer. Moreover, hepatocellular toxicity remains a barrier to administering larger sized NPs and increasing concentrations^37^. While larger nanoparticles are optimal for use with photothermal therapy^38^, smaller nanoparticles were more attractive for this study due to maximized absorption of characteristic X-rays to consequently optimize Auger emissions. Other studies have demonstrated that smaller nanoparticles deposit larger doses in their vicinity due to increased ionization events from a greater surface to volume ratio^39^. Smaller nanoparticles were also selected for this study because they have been shown to allow for increased uptake, longer circulation, and therefore improved tissue penetration^40,41^.

Photon interaction with DNA molecules can produce DNA DSBs^26^ while water radiolysis from photon-water interactions stimulates the production of ROS that target DNA to cause molecular injury^26,42^. The rapid course of intracellular events that occurs immediately following radiation was demonstrated using γ-H2AX with immunofluorescence microscopy^27,28^. Although the fluorescence intensity of γ-H2AX foci was higher in GNP-labeled, DTB irradiated HNCCs (Fig. 5a), there were fewer foci/nucleus than in cells irradiated with the STB (Fig. 5b). The high intensity and reduced foci in GNP-labeled cells may be attributed to the subcellular localization of short-range Auger emissions. Auger emissions deposit the greatest amount of ionization energy approximately 10 nm from a GNP, so the dose enhancement and resultant DSB is quite localized to the GNP itself^43^. As demonstrated by TEM (Fig. 1), GNPs were not uniformly distributed throughout the cytoplasm, yet the GNPs proximity to the nucleus was sufficient to induce DSBs (Fig. 5c). It is also important to consider that the FI of DSBs in GNP-labeled DTB irradiated cells was higher than DSBs with STBR. This may be an indication of the dose enhancement as a result of Auger emissions in the proximity of GNPs following DTBR.

While the zebrafish afforded many benefits to studying the unique interaction between GNPs and the DTB in HNCC, a significant limitation of the current model is that experimental processes do not reflect how this combination therapy would be administered to patients in clinic. Ideally, nanoparticles would be injected into a patient’s circulation and are expected to accumulate at the solid tumor site due to the enhanced permeability and retention effect^6,44,45^. The entire zebrafish larva was irradiated in this preclinical study, in contrast to a more focused region in the vicinity of a tumor, which would be the clinical approach. Furthermore, although single doses of 8 Gy are used in certain stereotactic ablative radiotherapy (SABR) treatment plans, conventional fractionation of 1.8 to 2 Gy per fraction remains the standard and was not employed in this study due to practical limitations in access to the radiation facility.

Despite these caveats, the study presents the first reported in vitro and in vivo biological responses to the novel DTBR combined with GNPs. With direction from the data we present in this work, future studies are aimed at further characterizing and optimizing GNP parameters in vitro and in vivo using functionalization, fluorescent markers, and confocal imaging. With targeted NPs, selectively boosting radiation dose to tumors which have traditionally been treated sub-optimally due to the dose constraints of the nearby critical organs may be possible. Although further pre-clinical studies are required to validate the efficacy and breadth of nanoparticle-mediated radiotherapy, these results provide promising data to support the use of the DTB with NPs as a potential treatment for HNC and other tumors located in close proximity to radiosensitive organs. This combination therapy allows for the administration of a lower dose that can be amplified by NPs to obtain similar efficacy as the current standard treatment but with a lower toxicity. Alternatively, it can provide an opportunity to escalate the dose to the tumor while minimizing normal tissue complication probability.

## Conclusion

This study presents the first reported in vitro and in vivo responses to the novel DTBR combined with GNPs. We have demonstrated the utility and potential of GNP-facilitated DTBR to decrease cell viability and tumorigenic potential of HNCC in vitro and in vivo. HNCCs demonstrated increased intensity of DSBs when labelled with GNPs and treated with DTBR. The high intensity, yet reduced quantity of DSBs in HNCCs may be attributed to the subcellular localization of short-range Auger emissions from the DTB. These results provide promising preclinical data to support further exploration of the DTB with NPs as a potential treatment for HNC and other anatomically challenging tumors. NP-enhanced DTBR has the potential to enhance radiotherapy delivery by improving the localization of radiation dose to the tumor volume. This advantage could be used either to escalate the tumor dose, thereby improving tumor control probability, or to minimize toxicity to adjacent organs and tissues, thus improving normal tissue complication probability. Since inadequate GNP uptake may be a barrier to effective DTBR, molecular targeting of NPs to increase distribution and duration in cells is predicted to selectively boost radiation dose to tumors which have traditionally been treated sub-optimally due to the dose constraints of nearby critical organs.

## Materials and methods

### Cell lines and cell culture

All cell lines were maintained in a sterile tissue culture incubator with 5% CO_2_ at 37 °C in 75 cm^2^ culture flasks media supplemented with Fetal Bovine Serum (Wisent) and Penicillin Streptomycin (Thermo-Fisher Scientific, Waltham MA, USA). FaDu hypopharyngeal carcinoma, HSC-3 oral squamous cell carcinoma (JCRB), and Detroit-562 pharyngeal carcinoma cell lines are HNCC lines, which were maintained in Eagle’s Minimum Essential Medium (EMEM). Panc1 pancreatic adenocarcinoma and A673 Ewing sarcoma cell lines were cultured in Dulbecco’s Modified Eagle’s Medium (DMEM). SK-N-AS neuroblastoma cells were cultured with DMEM supplemented with non-essential amino acids. All cell lines, except HSC-3 (JCRB) are from the American Type Culture Collection (ATCC).

### Nanoparticle labeling

Gold nanoparticles (GNPs, developed as “AuNPs”) were acquired from Nanoprobes (NY, USA) and reconstituted according to the manufacturer’s protocols. GNPs have a 15 nm core diameter, stabilized and capped by an organic, neutral, water-soluble ligand shell. The coating is composed of synthetic, hydroxylated hydrocarbon short polymers which coordinate to the gold surface via incorporated thiol (–SH) groups. No bio-functional groups were added to the GNPs. Approximately 1 × 10^6^ cells were labeled with 25 µM GNPs suspended in an appropriate volume of media 24 h prior to in vitro or in vivo experimentation. Control cells were not labelled with GNPs (NoNP).

### Transmission electron microscopy (TEM)

Transmission electron microscopy (TEM) was used to investigate the localization of GNPs. Cells were seeded in Petri plates and labeled with 25 µM GNPs for 24 h. Cells were fixed and scraped for further processing with TEM. Images were taken with a 120 kV JEOL 1230 TEM (80 kV) and captured using a Hamamatsu ORCA-HR digital camera.

### Radiation beam setup

Irradiation was performed on a TrueBeam^®^ (Varian Medical Systems, Palo Alto, CA) medical linear accelerator (LINAC). The custom 2.5 MV DTB is housed inside a TrueBeam LINAC^6^. The DTB is generated by accelerating electrons to 2.5 MeV and directing them towards a sintered diamond target, housed in the LINAC head carousel^6^. For the DTB, a polycarbonate filter (33 cm × 32 cm × 1 cm thick) was placed after the mylar window, in an effort to remove contaminant electrons. For comparison, a 6 MV standard target beam (STB) was used, with no additional filtration. In order to maintain a uniform dose of radiation for all samples, independent of beam type, samples were irradiated with fixed gantry (0°), and collimator (0°) angles, at a source-to-surface distance (SSD) of 74 cm from the tungsten target, defined by a 20 × 20 cm^2^ radiation field. Reference doses were calculated in accordance with the American Association of Physicists in Medicine (AAPM) task group report TG-51, for the 6 MV STB, and 2.5 MV DTB beamlines. After reference doses were established, the SSD was changed to 74 cm, and samples were placed surrounded by solid water. The 6 MV STB was operated in a non-clinical capacity and the lowest setting of 60 monitor units (MU) per minute was used (approximately 1.5 Gy/min). For the 2.5 MV DTB irradiation, we observed a dose rate of approximately 160 MU/min, corresponding to approximately 1.2 Gy/min.

### alamarBlue assay

The alamarBlue assay was used to measure in vitro cell viability. Cells were cultured in 96-well plates, incubated with GNPs, and irradiated with 8 Gy. alamarBlue (ThermoFisher) was added to each well at 3 days post-radiation (dpr) and the fluorescence of each well was measured using a Fluoroskan Ascent FL Microplate Fluorometer and Luminometer (Thermo-Fisher Scientific). The absorbance was measured at 570 nm with a baseline correction of 600 nm. Fluorescence fold change values were calculated.

### Colony formation assay (CFA)

Colony formation assays (CFAs) were used to measure the in vitro surviving fraction of cells. Cells were cultured in 6-well plates, labeled with GNPs, and irradiated with 8 Gy. At 24 h post radiation (hpr), serial dilutions from single cell suspensions were seeded. Plating efficiency (PE) was calculated for each cell line. At 10 dpr, cells were fixed with methanol and incubated with 1% (w/v) crystal violet. Digital brightfield images were captured, and colonies (colony ~ 50 cells) were counted using ImageJ software. The surviving fraction (SF) of cells was calculated [SF = number of colonies/(PE × number of cells seeded)].

### Zebrafish husbandry

Zebrafish (*Danio rerio*) were maintained according to standard protocols^43^. Adult zebrafish were kept at 28.5 °C on a 14-h light: 10-h dark schedule. Transparent double mutant *casper* zebrafish were used^46^. The use of zebrafish in this study was approved by the Dalhousie University Committee on Laboratory Animals (Protocol 19-051). All animal experiments were conducted in accordance with relevant institutional guidelines and regulations. Studies were carried out in compliance with the ARRIVE guidelines.

### Xenotransplantation (XT)

Cells were incubated with GNPs, harvested, labeled with CMTMR CellTracker Orange dye (Thermo-Fisher Scientific, Waltham MA, USA), and resuspended in media. Prior to injection, zebrafish larvae were anesthetized at 48 h post fertilization (hpf) with 0.090 mg/mL Tricaine. The PLI-100A Pico-injector microinjection system (Warner Systems, Hamden, CT) was used for XT injections. Anesthetized 48 hpf zebrafish larvae were arrayed in an injection plate and ~ 50–100 cells were injected into the yolk sac of each larva. At 12–24 h post injection (hpi), injected larvae were screened using a Zeiss V20 Axiocam 506 fluorescent microscope (Carl Zeiss, Wetzlar, Germany) for a uniform fluorescent cell bolus in the yolk sac and maintained at 35 °C for the remainder of the experiment.

### Larvae irradiation procedure

One day post-injection (dpi), approximately 30 positive larvae from the GNP and NoNP injected groups were placed into wells of a 6 well plate (with E3 medium-5 mM NaCl, 0.17 mM KCl, 0.4 mM CaCl_2_, and 0.16 mM MgSO_4_ supplemented with methylene blue (1 × 5–10% [v/v])). Plates were irradiated with 8 Gy, then returned to 35 °C until experimentation.

### In vivo proliferation-whole embryo dissociation

At 24 h post injection (hpi) and 48 h post radiation (hpr), 20 injected larvae (from NoNP and GNP) were euthanized. The larvae were dissociated into single-cell suspensions according to a previously established protocol^20,47^. Images of cell suspensions were taken using an inverted Axio Observer Z1 microscope (Carl Zeiss, Wetzlar, Germany). Images were captured at 24 hpi and 48 hpr and analyzed using ImageJ software (NIH, Bethesda, MD, USA) where relative fluorescent cell numbers were determined per larva.

### Flow cytometry

CellROX Deep Red (Thermo-Fisher Scientific, Waltham, MA, USA) was used to detect reactive oxygen species (ROS). Specifically, superoxide anions, hydroxyl radicals, and hydrogen peroxides were investigated. Cells were seeded in six-well plates, labeled with GNPs and irradiated. 12 h after 8 Gy treatment, cells were harvested. CellROX Deep Red reagent (Ex/Em 644/665 nm) and SYTOX Blue Dead Cell stain (Ex/Em 444/480 nm) were used to process samples. Flow cytometry was done using the BD FACSCanto flow cytometer (BD Biosciences, San Jose, CA), with R660 and V510 laser lines. Data were analyzed using BD FACSDiva Software. Gating strategy is outlined in Supplemental Fig. S6.

### Immunohistochemistry (IHC)

γ-H2AX (Phospho S139) monoclonal antibody was used to identify DNA double strand breaks. Cells were seeded on sterile 18 × 18 mm coverslips (Globe Scientific Inc., NJ, USA) in 6-well plates, labeled with GNPs and irradiated with 8 Gy. Cells were fixed 30 min after radiation, a mitotic time point selected for significant DSBs following radiation. Cells were fixed, permeabilized, washed, blocked, then incubated with phospho-histone H2AX (Ser139) (20E3) rabbit monoclonal antibody (Cell Signaling Technology) and anti-alpha tubulin [DM1A] microtubule marker (Alexa Fluor 488) mouse monoclonal antibody overnight. Following primary antibody incubation, samples were washed and incubated with donkey anti-rabbit polyclonal antibody (Alexa Fluor 647) and DAPI (4,6-Diamidino-2-Phenylindole, Dihydrochloride) (Invitrogen) for 1 h. Samples were washed, mounted, and imaged with the Zeiss LSM 710 laser scanning confocal microscope.

### IHC image analysis

Quantification of γ-H2AX staining was performed using a custom ImageJ (Fiji) macro. Regions of interest (ROI) for individual nuclei were generated, and area, integrated density of DAPI and γ-H2AX channels, and γ-H2AX foci count were measured.

### Statistical analysis

For alamarBlue assays, CFA, and XT assays, MiniTab 17 (MiniTab Software Inc., Pennsylvania, USA) was used to conduct statistical analyses. Significance between groups was tested using one-way analysis of variance (ANOVA) with a Tukey multiple comparisons test. For flow cytometry and IHC image analysis, differences between groups were tested using the Mann Whitney test. A p-value less than 0.05 was considered significant.

## Supplementary Information

Supplementary Figures.

## Data Availability

All data generated or analysed during this study are included in this published article (and its Supplementary Information files).
