# Supplementary material for: Radiation dose enhancement using gold nanoparticles with a diamond linear accelerator target: a multiple cell type analysis
Source: Sci Rep. 2022 Jan 28;12:1559. doi: 10.1038/s41598-022-05339-z (PMC8799734; doi:10.1038/s41598-022-05339-z)
Supplement: Supplementary file 1 — Supplementary Figures. [file 41598_2022_5339_MOESM1_ESM.pdf]

Supplemental Figures for

Radiation dose enhancement using gold nanoparticles with a diamond linear accelerator target - A multiple cell type analysis

Olivia Piccolo, John D. Lincoln, Nicole Melong, Benno C. Orr, Nicholas R. Fernandez, Jennifer Borsavage, Jason N. Berman, James Robar, Michael N. Ha

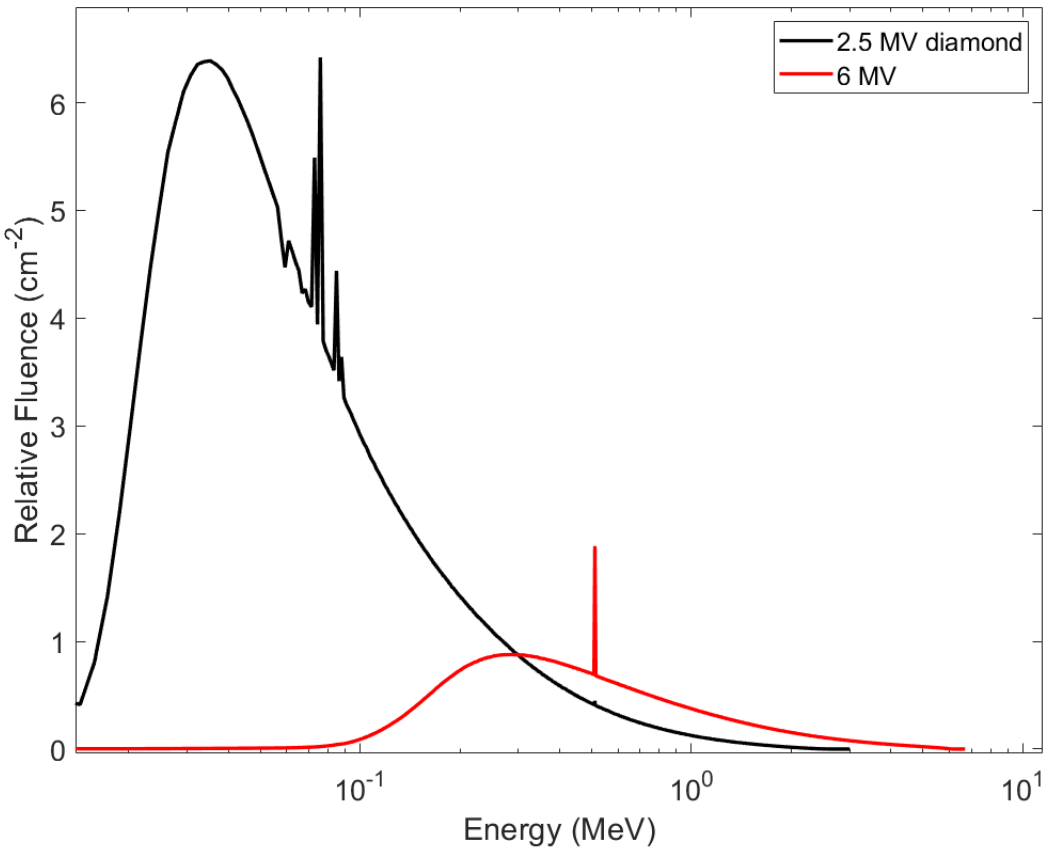

Supplemental Figure S1. Comparison between 2.5 MV diamond and 6 MV standard therapeutic energy spectra, demonstrating increased content of low-energy photons in the diamond target beam.

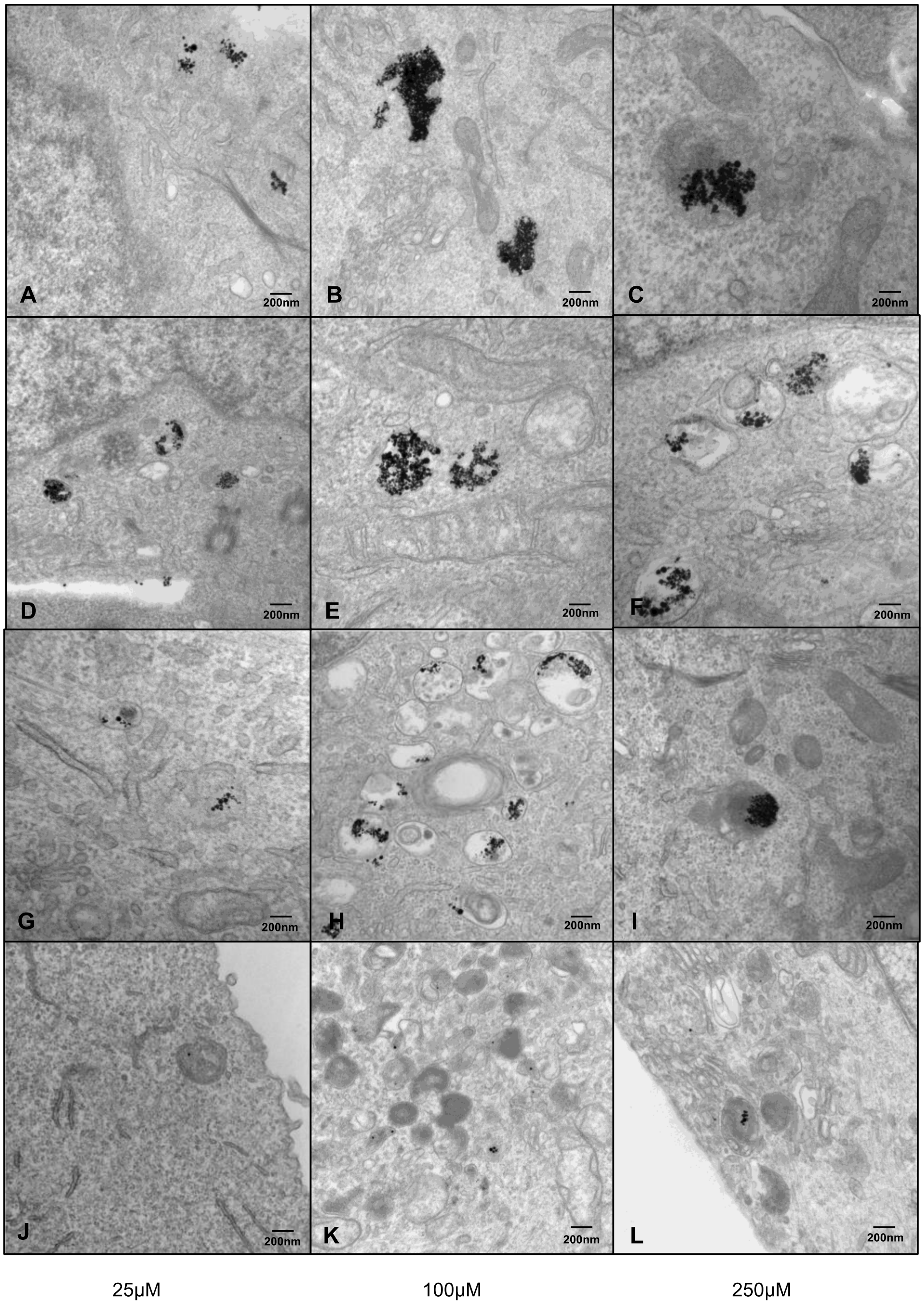

Supplemental Figure S2. **GNP uptake in cell lysosomes increases with higher concentrations of GNPs in HNCCs.** Representative images of **a)-c)** FaDu hypopharyngeal carcinoma cells; **d)-f)** HSC-3 hypopharyngeal carcinoma cells; **g)-i)** Detroit-562 pharyngeal carcinoma cells; and **j)-l)** Panc1 pancreatic adenocarcinoma cells labelled (from left to right) with 25 μM, 100 μM, and 250 μM GNPs. Cells were fixed ~36 hours after labelling with GNPs (approximate radiation timepoint) and images were captured with a transmission electron microscope (TEM). Acronym(s): GNP(s)- gold nanoparticle(s); HNCC- head and neck cancer cell.

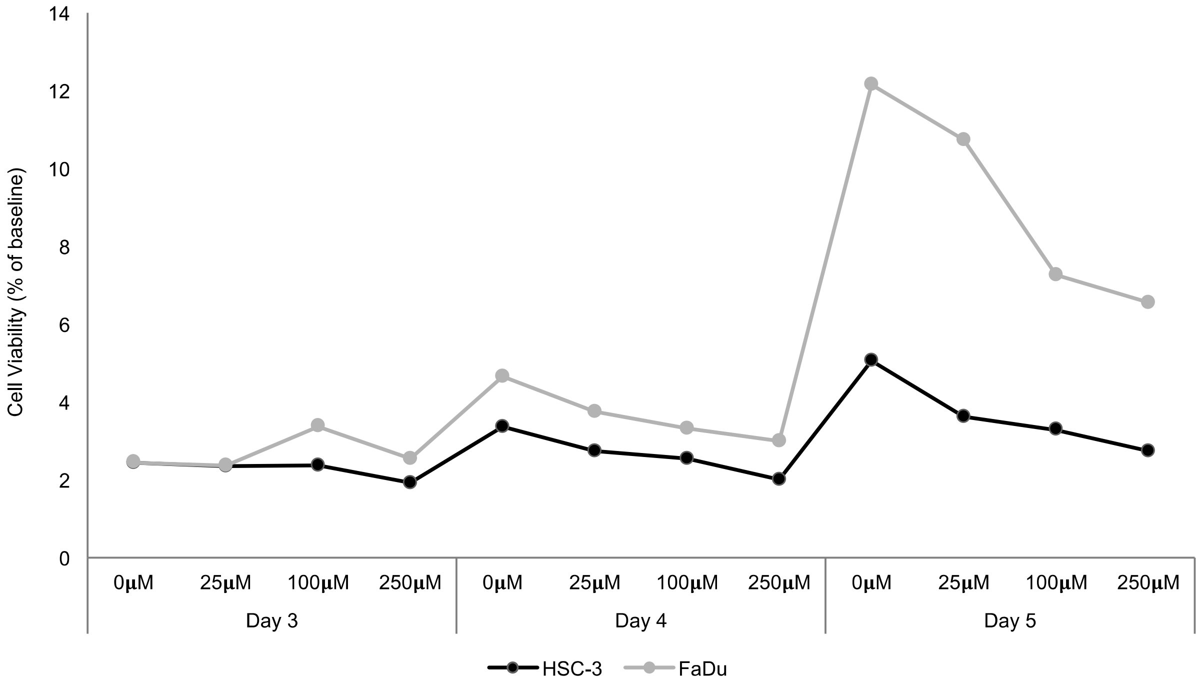

Supplemental Figure S3. GNPs decrease cell viability at higher concentrations. The Alamarblue viability assay was used to determine the levels of oxidative phosphorylation in FaDu and HSC- 3 cells labelled with increasing concentrations of GNPs. The fluorescent output of metabolically active cells was recorded after 24 h incubation with GNPs and cell viability was measured from days 3 to 5 after the baseline assessment. Viability of HSC-3 cells was significantly hindered at 5 days with 100 μM and 250 μM GNPs (p=0.022 and p=0.009, respectively). Cell viability values were made relative to baseline cells and are presented as the fold change means ± standard error of the mean. Significance between groups was tested using two-way analysis of variance (ANOVA) with a Tukey pairwise multiple comparisons test (n=3; 10 wells per group/replicate). Acronym(s): GNPs- gold nanoparticles.

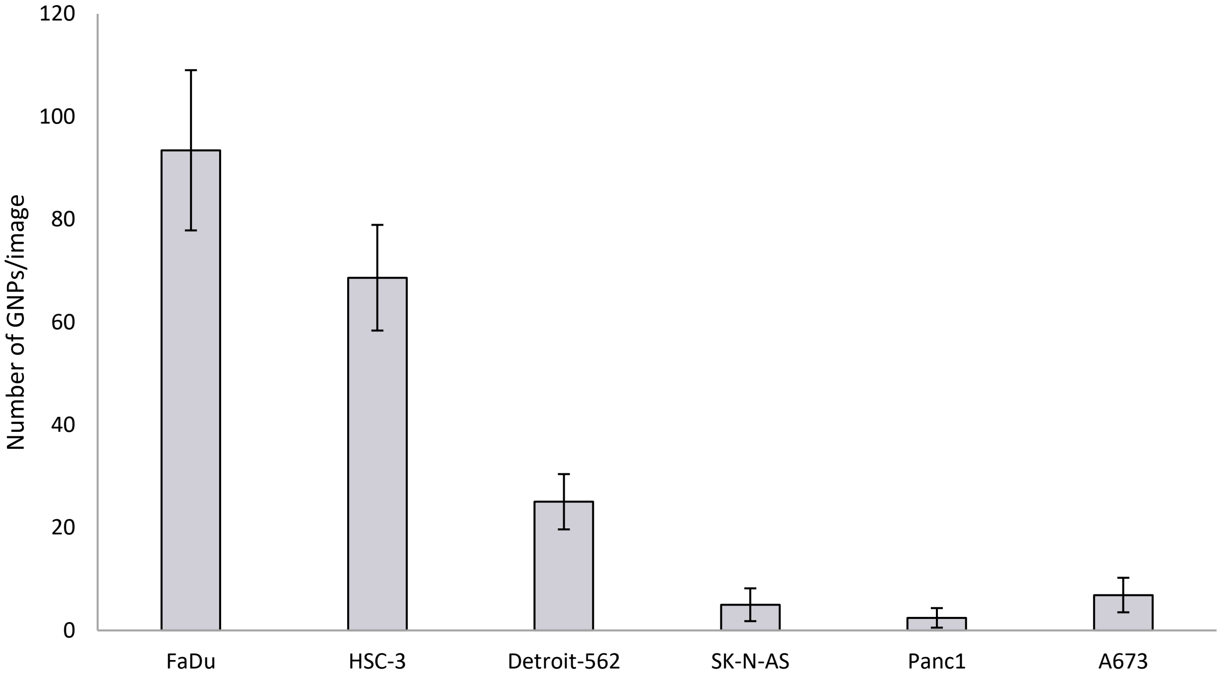

Supplemental figure S4. Diffusion of GNPs was highest in HNCC lines. Transmission Electron Microscope (TEM) images were taken of FaDu, HSC-3, Detroit-562, SK-N-AS, Panc1, and A673 cells labelled with 25 µM GNPs (n=45 for each cell line). Imaging was done in triplicate – 5 images were captured for each sample, with 3 samples of each cell line/replicate, and 3 replicates in total. Total number of GNPs was counted for each image, and the average number of GNPs in each image was calculated. Acronym(s): HNCC – head and neck cancer cell; GNPs- gold nanoparticles.

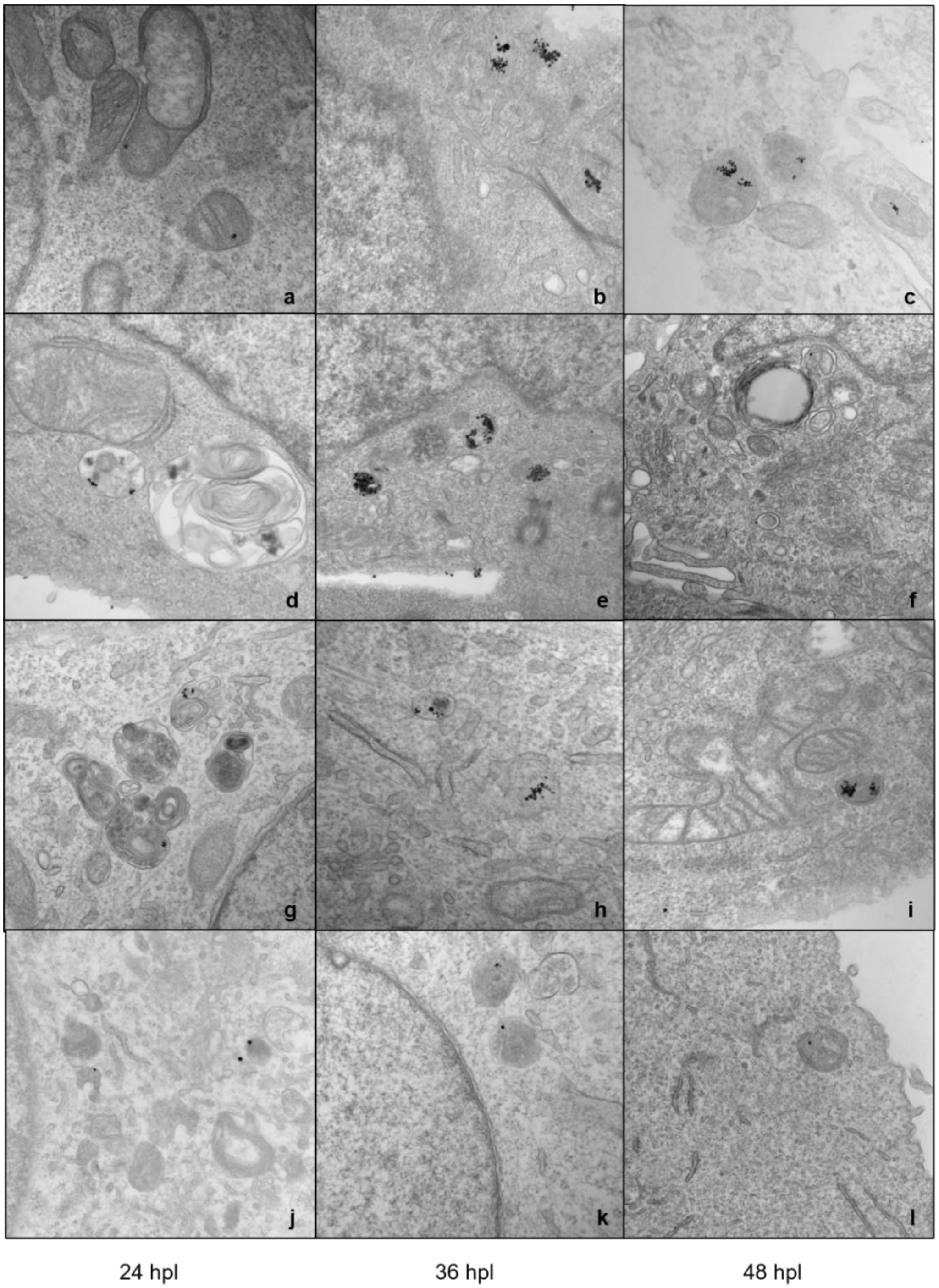

Supplemental Figure S5. **GNPs diffuse into cell lines at 24 hours post-labelling and demonstrate retention in cells 48 hours post-labelling.** Representative images of **a)-c)** FaDu hypopharyngeal carcinoma cells; **d)-f)** HSC-3 hypopharyngeal carcinoma cells; **g)-i)** Detroit-562 pharyngeal carcinoma cells; and **j)-l)** Panc1 pancreatic adenocarcinoma cells imaged (from left to right) at 24, 36, and 48 hpl. Cells were fixed at appropriate timepoint and images were captured with a transmission electron microscope (TEM). Acronym(s): GNPs- gold nanoparticles; hpl- hours post labelling.

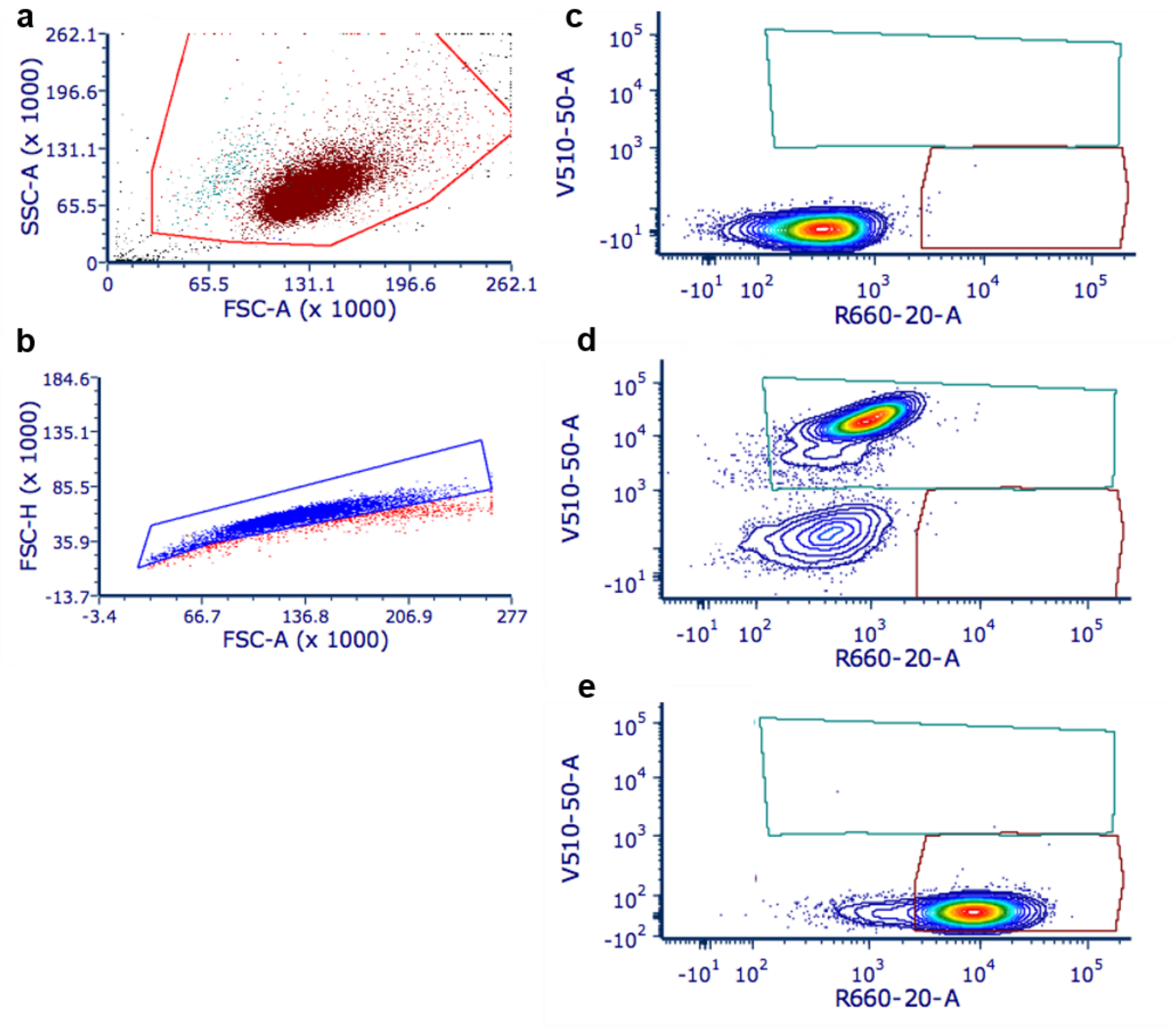

Supplemental Figure S6. Flow cytometry gating strategies for the detection of ROS and cell death. a) General gating strategy to identify living and dead cells in sample and eliminate cell debris. **b)** Doublet discrimination gating strategy. **c)** Unstained control cells. **d)** Gating strategy to identify SYTOX + dead cells, representative plot from heat-killed SYTOX + control cells. **e)** Gating strategy to identify CellROX + cells, representative plot from cells treated with tert-butyl hydrogen peroxide to produce reactive oxygen species. Acronym(s): ROS- reactive oxygen species.
